# Anti-thymocyte globulin-based treatment frequently leads to enduring treatment success in both old and young adult patients with aplastic anaemia: a real-world analysis from the Dutch aplastic anaemia registry

**DOI:** 10.1007/s00277-026-06743-5

**Published:** 2026-01-22

**Authors:** C. J. M. Halkes, E. A. S. Koster, E. J. M. Bogers, F. C. J. I. Heubel-Moenen, L. G. M. Daenen, S. K. Klein, S. M. C. Langemeijer, E. Nur, M. H. G. Raaijmakers, T. J. F. Snijders, J. M. L. Tjon, L. C. de Wreede

**Affiliations:** 1 Department of Haematology, Leiden University Medical Centre, Leiden, The Netherlands; 2 Department of Haematology, Maastricht University Medical Centre+, Maastricht, the Netherlands; 3 Department of Haematology, University Medical Centre Utrecht, Utrecht, The Netherlands; 4 Department of Haematology, University Medical Centre Groningen, University of Groningen, Groningen, The Netherlands; 5 Department of Haematology, Radboudumc, Nijmegen, The Netherlands; 6 Department of Haematology, Amsterdam UMC, University of Amsterdam, Amsterdam, The Netherlands; 7 Department of blood cell research, Sanquin Research, Amsterdam, The Netherlands; 8 Department of Haematology, Erasmus MC Cancer Institute, Rotterdam, the Netherlands; 9 Department of Haematology, Medisch Spectrum Twente, Enschede, The Netherlands; 10 Department of Biomedical Data Sciences, Leiden University Medical Center, Leiden, The Netherlands

**Keywords:** Aplastic anemia, Long-term outcomes, Immune suppressive treatment, Anti-Thymocyte globulin, Bone marrow failure

## Abstract

Discussion remains concerning the safety and tolerability of anti-thymocyte globulin (ATG)-based immunosuppressive therapy (IST) in older patients with aplastic anaemia (AA). Using data of 127 consecutive patients from the Dutch adult AA registry, we evaluated long-term treatment success of standard ATG-based IST as first-line treatment with a multi-state model. Only one death was potentially associated with ATG. Overall survival at 5 years was 79%. We defined Transplantation-, Treatment- and Disease-Free Survival (TT-DFS) as the ultimate success of this treatment. This means that a patient is alive, is currently transfusion-independent without having received an allogeneic stem cell transplantation, has not developed AML or MDS, and has stopped all medication for AA. The probability of TT-DFS was 42% at 5 years after start of IST. In patients younger than 40 years (*n* = 36) and in patients aged 60 or above (*n* = 53), this was 58% and 34%, respectively. Older age, more severe AA and absence of a PNH-clone of ≥ 1% all reduced the likelihood of reaching TT-DFS. These analyses on unselected nationwide data indicate that ATG-based IST is effective and safe also in older patients. They suggest that age, AA severity and presence of a PNH-clone should be taken into account when considering this treatment in older patients.

**Supplementary Information:**

The online version contains supplementary material available at 10.1007/s00277-026-06743-5.

## Introduction

 Acquired aplastic anaemia (AA) is a rare haematological disease characterized by pancytopenia and a hypocellular bone marrow. The exact pathogenesis is unknown but most likely involves an auto-immune reaction [1, 2]. The preferred first-line treatment in the majority of adult patients is intensive immunosuppressive therapy (IST) based on anti-thymocyte globulin (ATG) and cyclosporine (CsA) with or without Eltrombopag [3]. As this treatment is associated with an increased risk of acute cardiac toxicity and infusion reactions and conflicting results are published concerning its effectivity in patients aged 60 years or older, there is debate whether ATG-based IST should be offered as preferred first-line treatment to older AA patients instead of a less intensive treatment [3–6].

Recently, an analysis based on two single-centre clinical trials which ran between 2005 and 2022, showed that acute toxicity after IST, response rate at six months and overall survival (OS) were similar in responding patients aged below 60 and of at least 60 years [5]. The authors concluded that ATG-based IST should be the preferred first-line treatment in all AA patients aged 60 and older. In contrast, another analysis on patients treated between 1976 and 2024 in 4 tertiary hematologic centers showed an inferior response rate after ATG-based IST in AA patients aged 60 and older compared to patients younger than 60. The authors concluded that older patients had an unfavorable risk/benefit ratio for ATG-based therapy and that this treatment should only be offered to AA patients younger than 60 and to patients between 60 and 65 years without comorbidities [6].

When considering ATG-based IST as first-line treatment, not only the risk of severe side effects in older patients with comorbidities, but also the probability to achieve an enduring response should be taken into account. Hematological recovery at six months after start of IST is commonly used as a measure for treatment success of IST. Younger age, non-severe AA and the presence of a glycosyl phosphatidylinositol (GPI)-deficient cell clone (Paroxysmal Nocturnal Hemoglobinuria (PNH)-clone) at time of diagnosis are associated with reaching this short-term treatment success in several studies [7–9]. Long-term treatment success and survival after IST in patients with AA is hampered by disease related mortality due to infections or bleedings, toxicity of second-line treatments like allogeneic stem cell transplantation (alloSCT) and the development of other bone marrow diseases like MDS or AML. The most common treatment failure is relapse of AA, after which a renewed response can often be achieved by reintroduction of IST. These complex dynamics that are all relevant for achieving and maintaining long-term IST treatment success cannot be captured by the fixed 6-month timepoint for haematological response and long-term OS.

Therefore, we propose a comprehensive measure of treatment success over time after ATG-based IST for AA. The main component of this measure is Disease-free survival (DFS): being alive and currently transfusion-independent without having developed other bone marrow diseases like MDS or AML. We consider this the broad aim of ATG-based IST. DFS should preferably be achieved without needing an alloSCT as second-line treatment: Transplantation-free DFS (T-DFS). The ultimate aim of IST is to stop all medication after achieving a response, leading to the endpoint Transplantation- and Treatment-free DFS (TT-DFS). All these endpoints are dynamic, i.e. during time since start of treatment they are continuously updated when responses are gained, lost and regained or other relevant events take place. These complex endpoints can be assessed by multi-state models, which can also serve to investigate the impact of risk factors on different steps of the recovery/disease process. We used DFS, T-DFS and TT-DFS to evaluate treatment success in patients from the Dutch adult aplastic anaemia registry who were treated uniformly with horse-derived ATG (ATGAM)-based IST. This registry describes a unique real-world nationwide cohort containing data of 144 consecutive adult AA patients who were treated with ATGAM-based IST in the Netherlands between 2012 and 2021.

## Materials and methods

### Patients and treatment

The Dutch national AA registry is a population-based registry in which data is collected from all consecutive patients in the Netherlands who received ATGAM in combination with CsA as first-line treatment for AA according to the national guideline [10] (Supplemental Methods). Treatment is given in a limited number of Dutch hospitals, that all contribute data to the registry (Supplemental Table 1). Completeness of patient cohorts per hospital was checked using delivery data of ATGAM to the hospital pharmacies. Clinical data and laboratory results were collected by the treating haematologists or local data managers at baseline and at regular intervals after start of ATGAM and checked by a central data manager. The Institutional review board of the Leiden University Medical Centre approved data collection and analysis (protocol nr. C13.014). The dataset was closed on July 1 st 2022.

## Definitions and endpoints

AA diagnosis was classified according to the Camitta criteria [11]. Transfusion independency was defined as having been free of transfusion for at least 4 weeks. Non-transplant therapy was defined as any treatment to improve haematopoiesis in AA except alloSCT. This could include multiple courses of ATG, CsA, other immunosuppressive drugs or drugs with another working mechanism like Eltrombopag or Danazol. Disease-free survival was defined as being alive and currently transfusion-independent without having started treatment for a secondary bone marrow disease, i.e., AML or MDS. Transplantation-free DFS was defined as DFS without having received an alloSCT. Treatment- and Transplantation-free DFS was defined as T-DFS without having received any non-transplant therapy for at least 2 weeks. The primary endpoint of this analysis was the probability of TT-DFS until 5 years after start of ATGAM. Secondary endpoints were the cumulative incidence of achieving transfusion independency and probabilities of DFS, T-DFS and OS.

### Statistical analysis

OS from start of first IST with 95% confidence intervals (95%-CI) was calculated using the Kaplan-Meier method. Follow-up was quantified using the reverse Kaplan-Meier method which censors patients when they die [12]. The cumulative incidence of achieving transfusion independency after first-line treatment was estimated using a competing risks model with death and start of second-line treatment or treatment for AML or MDS as competing events. The cumulative incidence of achieving transfusion independency regardless of the number of treatments was estimated using a competing risks model with death and treatment for AML or MDS as competing events. To estimate DFS, T-DFS and TT-DFS after IST and to identify which factors influence the treatment response, a Markov time-inhomogeneous multi-state model was constructed. In a multi-state model patients transit between states at the occurrence of clinical events. Each transition hazard can either be estimated without taking covariates into account (non-parametrically) or analysed by means of a transition-specific Cox proportional hazards model (semi-parametric approach). The baseline hazards and the hazard ratios (HRs) are the building blocks for the calculation of the transition probabilities, which represent the probabilities of being in each of the states over time [13, 14].

**Fig. 1:**
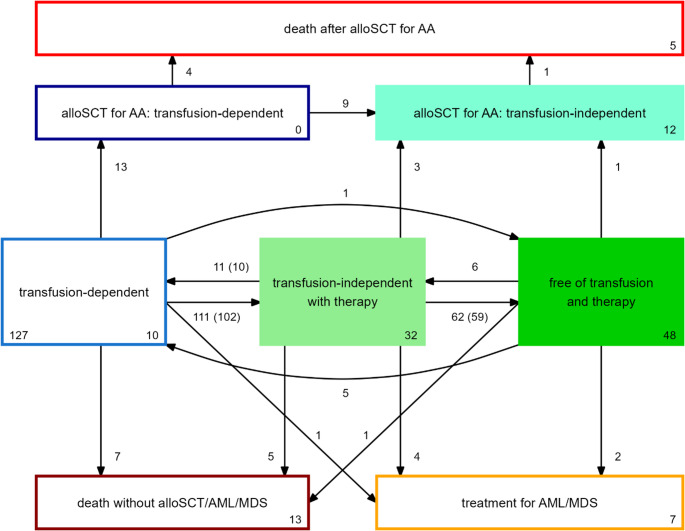
Structure of the multi-state model. Boxes represent states, arrows transitions. All patients start in the state ‘transfusion-dependent’. The number in the left corner of this box shows the number of patients included in the non-parametric model. The numbers at the bottom right corner of the boxes show the numbers of patients who were in that state at the end of their follow-up. The numbers next to the arrows show the numbers of transitions that were observed during follow-up. A few patients made a transition multiple times during their follow-up. For the transitions for which this was the case, the numbers inside the brackets show the numbers of unique patients who made these transitions. TT-DFS equals the probability of being in the state ‘free of transfusion and therapy’ (dark green), T-DFS the probability of being either in this state or in the state ‘transfusion-independent with therapy’ (light green), DFS the probability of being in any of the three filled states

The structure of the model is shown in Fig. 1. Starting state and time of the model is the start of the IST (ATGAM and Cyclosporine). As all patients are also transfusion-dependent at that time, this state is called ‘transfusion-dependent’. If a patient has been transfusion-independent for 4 weeks, this patient will move to the state ‘transfusion-independent with therapy’ after these 4 weeks. If a patient in this state has stopped all non-transplant therapy for 2 weeks and remains transfusion-independent, the patient will move to the state ‘free of transfusion and therapy’. Transfusion-independent patients who become transfusion-dependent and patients who have to restart non-transplant treatment will move to the appropriate states. From these three states, patients can experience three failure events: requirement of alloSCT, start of treatment for another bone marrow disease (AML or MDS) and death. The latter two are absorbing states, implicating that patients can never leave the state. AlloSCT is split into transfusion dependency after alloSCT, transfusion independency after alloSCT and death after alloSCT to evaluate the outcome after alloSCT as non-first-line treatment.

The effects of patient age, presence of a PNH-clone and severity of the AA on the hazards of several transitions of the multi-state model were estimated using transition-specific Cox models (Supplemental Methods). To translate the obtained HRs into clinically relevant measures, we calculated multi-state model-based outcomes for reference patients with different baseline characteristics. This shows the impact of age, the presence of a PNH-clone and the severity of the disease on T-DFS and TT-DFS.

Confidence intervals for DFS and T-DFS, which are combinations of multiple states, were calculated based on the estimated variance-covariance matrix of the transition probabilities [14].

## Software

All analyses were performed in R version 4.5.0 using the packages survival [15], prodlim [16], mstate [14], ggplot2 [17], and ggh4x [18].

## Results

### The majority of AA patients become transfusion-independent after IST

In total, 144 patients who received ATGAM-based IST between 2012 and 2021 were included in the Dutch AA registry. Seventeen patients received IST with the addition of Eltrombopag as first-line treatment in the prospective randomized EBMT RACE study [9]. To preserve a uniform treatment cohort we excluded these 17 patients. Analyses were performed on the remaining 127 patients.

Baseline characteristics of the patients are shown in Table 1. The median follow-up time was 56 months (interquartile range 25–80). Figure 1 shows the number of transitions between the different states. At start of IST, all 127 patients were transfusion-dependent. The cumulative incidence of achieving transfusion independency without any second-line treatment was 64% (95%-CI 55–72) at 1 year after start of ATGAM and CsA (Supplemental Fig. 1). For patients who did not become transfusion-independent, second-line treatment was Eltrombopag in 21 patients (of whom 15 became transfusion-independent), rabbit-derived ATG (Thymoglobulin) in 7 patients (of whom 3 became transfusion-independent) and 8 patients received an alloSCT. See Supplemental Fig. 2 for the respective third- and fourth-line therapies given after ATGAM with CsA. The cumulative incidence of becoming transfusion-independent regardless of the number of treatments was 88% (95%-CI 82–94) at 2 years after start of IST (Supplemental Fig. 1).

**Table 1:** Baseline characteristics of the 127 patients with ATGAM-based IST as first-line treatment

Median Age (range)Age category- no (%)	54 (18–79) years
Age 18–39 years	36 (28%)
Age 40–59 years	38 (30%)
Age ≥ 60 years	53 (42%)
Sex- no (%) Male Female	81 (64%) 46 (36%)
Disease severity- no(%) Non-severe Severe Very severe	42 (33%) 54 (43%) 31 (24%)
PNH-clone at diagnosis-no (%) < 1% ≥ 1% Missing	62 (55%) 50 (45%) 15
Cytogenetic abnormalities- no (%) Normal Abnormal karyotype* Karyotype analysis failed Missing	75/88 (85) 8/88 (9) 5/88 (6) 39
Median time between diagnosis and start ATGAM (range)**	1.6 (0.1–191) months

* Abnormal karyotype included loss of Y chromosome (6 patients), −13q (1 patient) and extra marker chromosome of unknown origin (1 patient)

** Time of diagnosis not known in 13 patients

### Low treatment-related mortality after ATG-based IST

Twenty-one patients died within 5 years after start of ATG (5 after alloSCT as non-first-line treatment for AA (median time between start IST and death 12 months) and 3 due to AML or MDS (median time between start IST and death 45 months)). Seven patients died due to cytopenia-related complications after a median time of 32 months (causes of death: haemorrhage, pneumonia or refractory aplastic anaemia). One patient (age 77 years) died due to cardiac failure ten days after start of IST, which could potentially have been associated with a newly diagnosed atrial fibrillation. All causes of death in the first 5 years after start of IST are shown in the Supplemental Table 2.

### At 5 years, Overall Survival is 79% and 42% of patients are Transplantation-, Treatment- and Disease-free

**Fig. 2:**
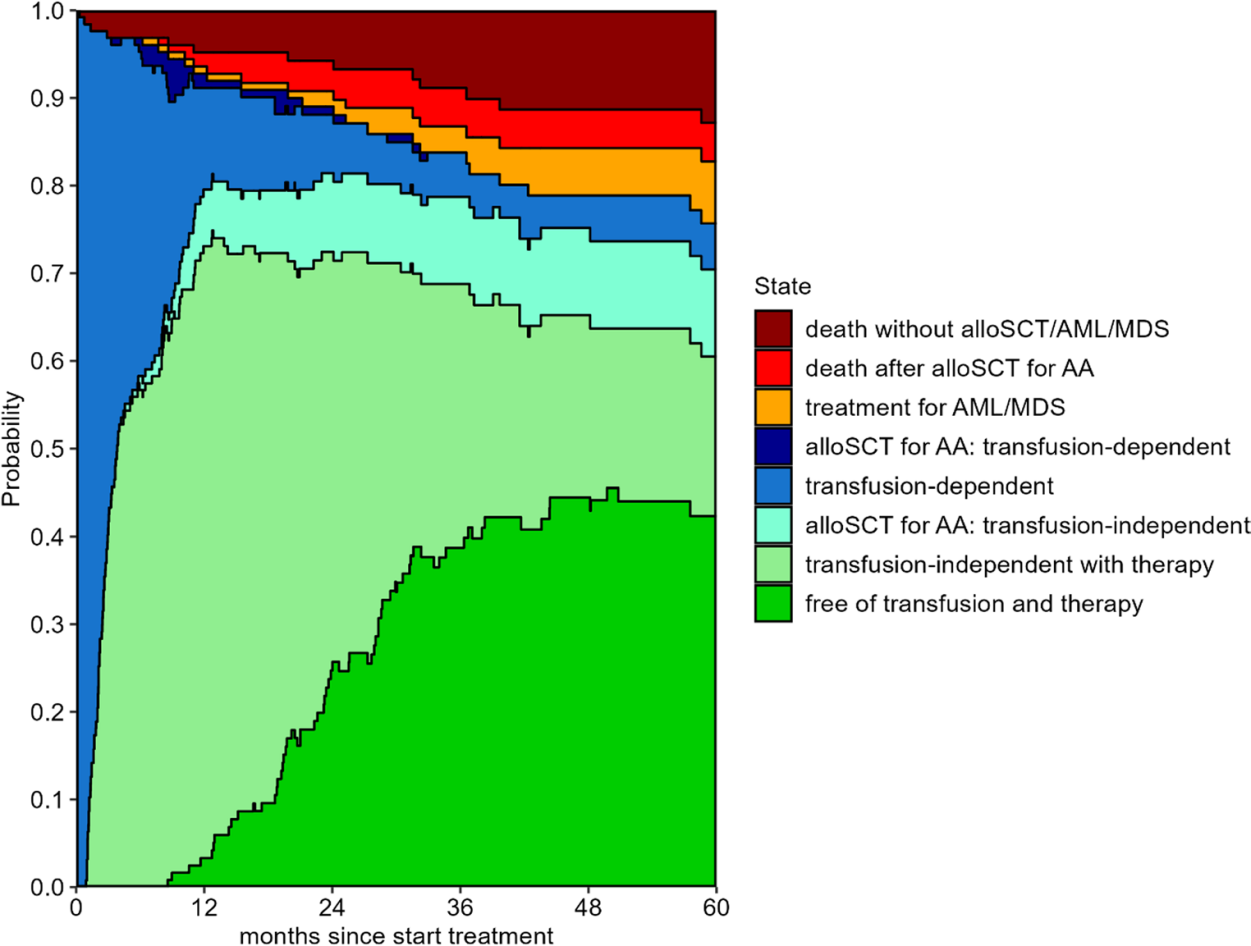
Outcomes after start of IST. Stacked transition probabilities from state ‘transfusion-dependent with therapy’: the difference between two adjacent curves represents the probability of being in the corresponding state. The probability of being in the state ‘free of transfusion and therapy’ represents the TT-DFS, the probability of being in this state or in the state ‘transfusion-independent with therapy’ the T-DFS. DFS is the sum of these two states and ‘alloSCT for AA: transfusion-independent’. At 5 years after alloSCT, the probabilities of DFS, T-DFS and TT-DFS were 70% (95%-CI 61–81), 60% (95%-CI 51–71) and 42% (95%-CI 33–54), respectively

**Fig. 3:**
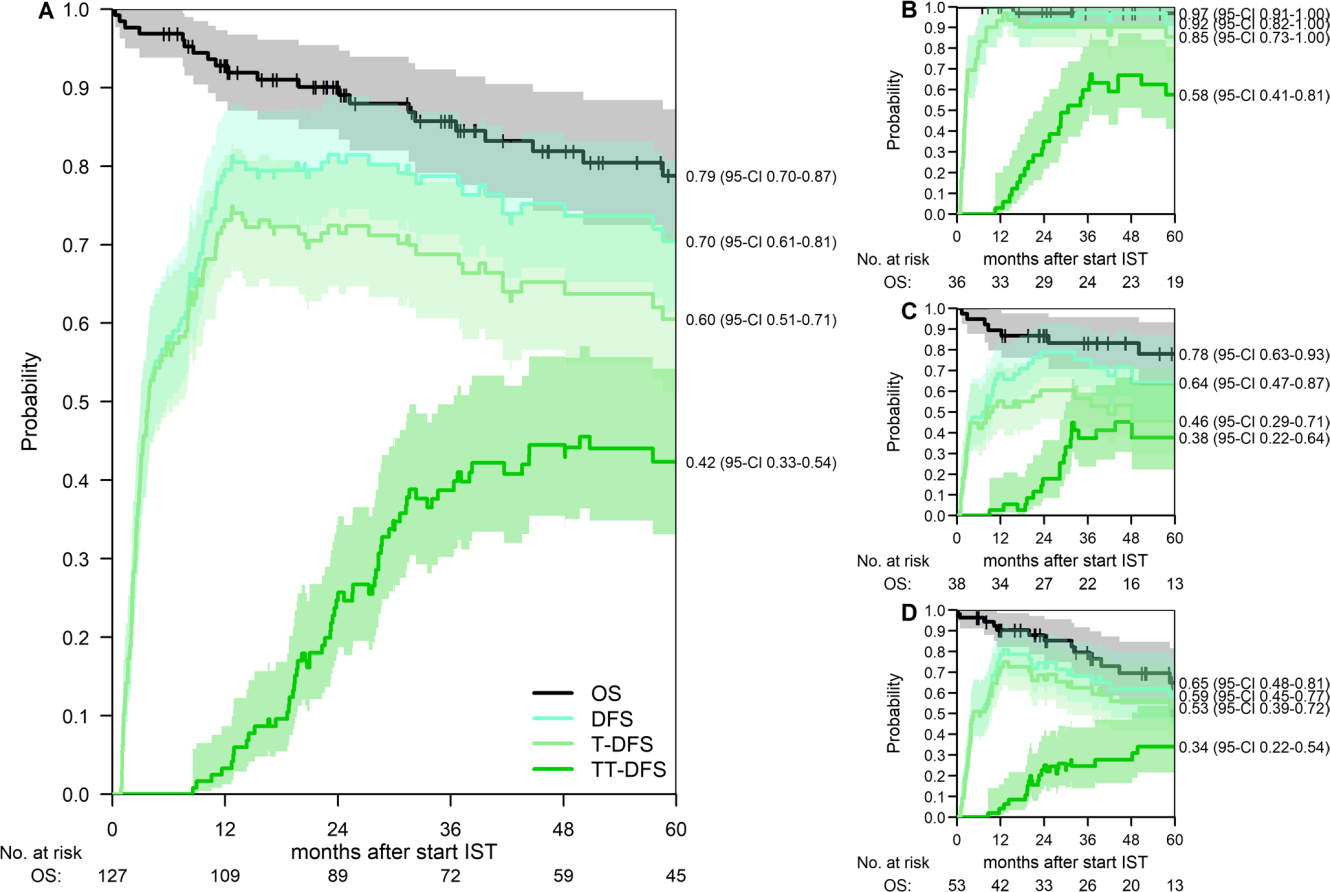
OS, DFS, T-DFS and TT-DFS. Probabilities of Overall Survival, Disease-free survival, Transplantation- and Disease-free survival and Transplantation-, Treatment- and Disease-free survival with associated 95% confidence intervals for the total cohort (A) and stratified by age group: 18–39 years (B), 40–59 years (C) and 60 years or older (D). The 5-year probabilities are stated next to the panels

Figure 2 shows the outcomes for patients during the first 5 years after start of first-line IST. Most patients became transfusion-independent during the first year and from the first year on, patients were also able to stop their medication for AA. Five years after start of IST, 70% (95%-CI 61–81) of the patients were transfusion-independent and had not developed MDS or AML (DFS). OS was 79% (95% CI 70–87%, Transplantation-free DFS (T-DFS) was 60% (95%-CI 51–71) and the Transplantation- and Treatment-free DFS (TT-DFS) was 42% (95%-CI 33–54) (Fig. 3A**)**. Figure 3B-D show OS, DFS, T-DFS and TT-DFS per age category. Patients aged 18–39 years at start of treatment had a 5-year OS of 97% (95%-CI 91–100), and a TT-DFS of 58% (95%-CI 41–81). For patients aged 40–59, the 5-year OS and TT-free DFS were 78% (95%-CI 63–93) and 38% (95%-CI 22–64), respectively. For patients of 60 years and older, the 5-year OS and TT-free DFS were 65% (95%-CI 48–81) and 34% (95%-CI 22–54), respectively.

## Age, disease severity and concomitant PNH-clone at diagnosis predict TT-DFS

**Fig. 4:**
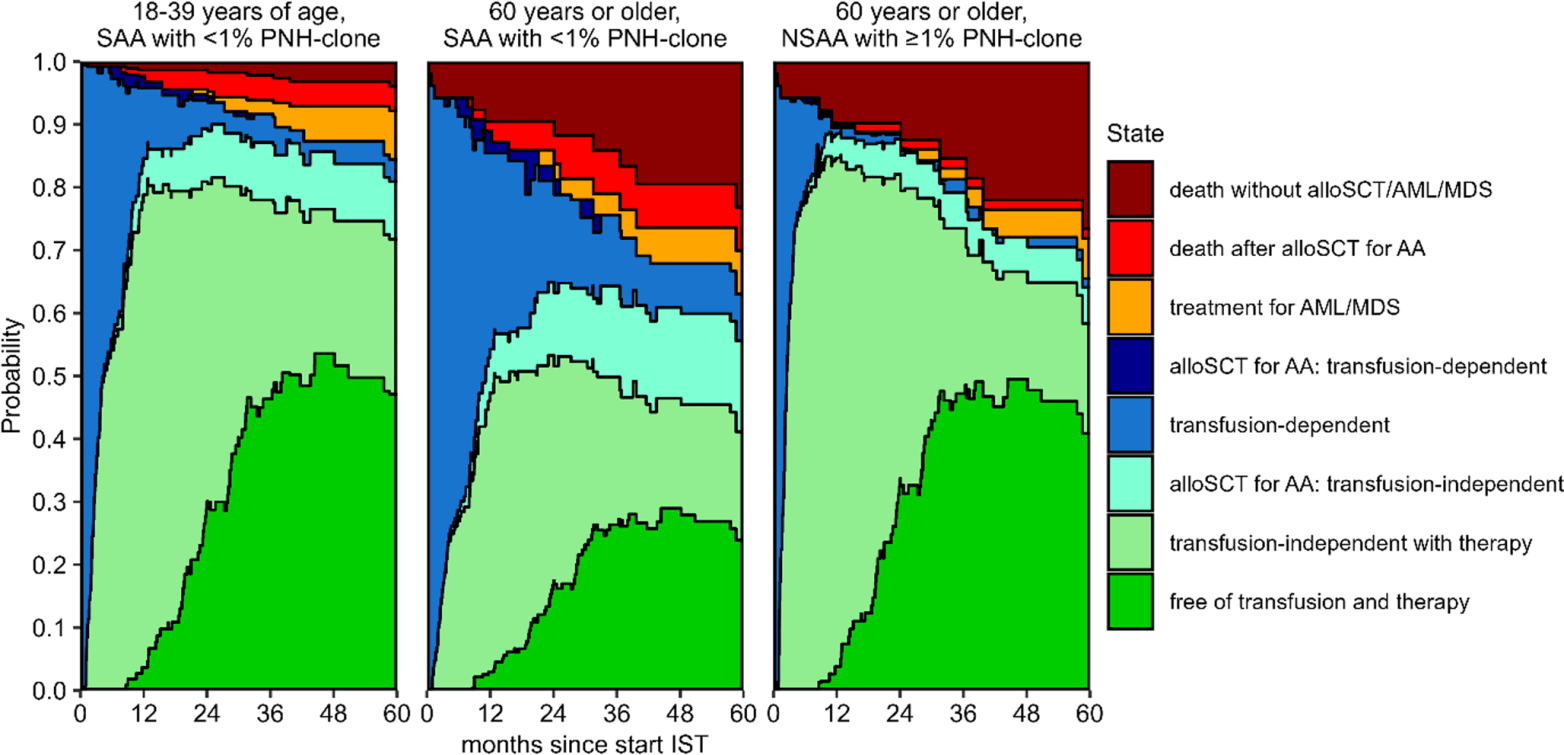
Model-based outcomes for three reference patients with different characteristics. These are based on the multi-state model in Fig. 1 and the transition-specific Cox models in Table 2. The difference between two adjacent curves represents the probability of being in the corresponding state. Supplemental Fig. 3 and Supplemental Table 3 show the model-based outcomes for all possible combinations of characteristics

Patients with a PNH-clone (> 1%) at diagnosis had a HR of 2.2 (95%-CI 1.4–3.4) to become transfusion-independent during AA treatment compared to patients without a PNH-clone. Patients aged 40–59 and older than 59 were less likely to become transfusion-independent compared to younger patients (HR 0.4 [95%-CI 0.2–0.7] and 0.4 [95%-CI 0.3–0.7], respectively). The same was observed for patients with (very) severe AA compared to patients with non-severe AA (HR for severe AA 0.4 [95%-CI 0.3–0.7] and for very severe AA 0.5 [95%-CI 0.3–0.8]). None of these risk factors had a significant effect on the likelihood of stopping all non-transplant treatments after having become transfusion-independent. Patients aged 60 or older were more likely to die (without alloSCT or treatment for AML or MDS) compared to patients younger than 60 (HR 7.3, 95%-CI 1.5–34.3) (Table 2). To show the impact of age, presence of a PNH-clone and disease severity on the different outcomes, we calculated model- based prognoses for reference patients with different baseline characteristics. Figure 4 shows the model-based outcomes after IST for a reference patient with severe aplastic anaemia who is < 40 years old and has no PNH-clone, a patient of 60 years or older with the same characteristics, and a patient of 60 years or older with non-severe AA and a PNH-clone of ≥ 1.0% at baseline. Five years after the start of IST, the model-based TT-DFS was 47% (95%-CI 33–68) for the young patient and 24% (95%-CI 14–41) for the older patient with bad characteristics, and 41% (95%-CI 26–65) for the older patient with NSAA and a PNH clone. Model-based outcomes for all 18 possible reference patients (based on age, disease severity and presence of PNH-clone at diagnosis) are shown in Supplemental Fig. 3 and Supplemental Table 3.

**Table 2:** Prognostic factors for becoming transfusion-independent with only non-transplant therapy, becoming free of transfusion and therapy, or dying without event

Transition	Factor	HR (95%-CI)	*P*-value
**Transfusion-dependent** ➜ **Free of transfusion with therapy**	PNH-clone: ≥1% vs. < 1%	2.19 (1.42–3.40)	< 0.001
	Age: 40–59 vs. 18–39 years	0.41 (0.24–0.69)	< 0.001
	Age: 60 + vs. 18–39 years	0.43 (0.26–0.71)	< 0.001
	Severity: SAA vs. NSAA	0.42 (0.27–0.67)	< 0.001
	Severity: VSAA vs. NSAA	0.46 (0.27–0.80)	0.005
**Free of transfusion with non-transplant therapy** ➜ **Free of transfusion and therapy**	PNH-clone: ≥1% vs. < 1%	1.12 (0.65–1.93)	0.69
	Age: 40–59 vs. 18–39 years	0.85 (0.43–1.70)	0.65
	Age: 60 + vs. 18–39 years	0.84 (0.45–1.58)	0.59
	Severity: SAA vs. NSAA	0.83 (0.43–1.59)	0.57
	Severity: VSAA vs. NSAA	0.99 (0.48–2.02)	0.98
**Transfusion-dependent**,** Free of transfusion with therapy or Free of transfusion and therapy** ➜ **Death without alloSCT/AML/MDS**	Age: 60 + vs. 18–59 years	7.28 (1.55–34.32)	0.01

Cox proportional hazards models for the transition from ‘transfusion-dependent’ to ‘transfusion-independent with therapy’, for the transition from ‘transfusion-independent with therapy’ to ‘free of transfusion and therapy’ and for the combined transitions to ‘death without alloSCT/AML/MDS’. Based on complete case analysis (*n* = 112, *n* = 91 and *n* = 127 for the three models, respectively). Age is determined at time of start IST

*HR* hazard ratio, 95%-*CI* 95% confidence interval, *SAA* severe aplastic anaemia, *NSAA* non-severe aplastic anaemia, *VSAA* very severe aplastic anaemia

## Discussion

This study is based on real-world data from a unique cohort of unselected adult AA patients who were treated with standard first-line IST in the Netherlands between 2012 and 2021. These patients had a 5-year overall survival of 79% (95%-CI 70–87), which is comparable to outcomes reported in Phase II studies [19, 20]. We showed that after 5 years 60% (95%-CI 51–71) of the patients were alive and transfusion-independent, without MDS or AML and without having received an alloSCT for AA. At that time, 42% (95%-CI 33–54) of the patients were alive and transfusion-independent without needing medication to treat AA, without MDS or AML and without having received an alloSCT for AA, which we consider as ultimate treatment success. We showed that age, disease severity and the presence of a PNH-clone are associated with treatment success.

Based on data from two prospective clinical trials from the NIH, Prabahran concluded that ATG-based IST is safe and effective in older patients with AA and that this should be the preferred first-line treatment in these patients [5]. We confirm these findings in a real-world cohort showing an OS at 5 years of 65% in patients aged 60 years and older. In the NIH analysis, the overall response (OR) rate at 6 months in these older patients was comparable to the OR in younger patients (72% and 73%, respectively). For older patients, the relapse rate was 71% and the cumulative incidence of high-risk clonal evolution was 17%. This raises the question whether the hematological responses in older patients are durable. If a majority of the older patients with a response to ATGAM-based IST suffer a relapse, develop high-risk clonal evolution or need an alloSCT as second-line treatment, the long-term success of this treatment would not be very satisfying for this patient group. The use of cumulative incidences to quantify response and different causes of failure to IST does not give information about the percentage of patients who have a long-term response after first-line IST without encountering one of these events. We advocate the use of multi-state modeling to deal with recovery after relapse and to show changes in patients’ states over time. This makes it possible to analyze dynamic outcomes like DFS including transfusion dependency, DFS without the need for an alloSCT (T-DFS) and T-DFS without need of ongoing medication (TT-DFS). Figure 1 shows that transitions between these dynamic states (transfusion-dependent, transfusion-independent with therapy and transfusion-independent without therapy) occur frequently and Fig. 2 shows that only a small minority of patients is transfusion-dependent at 5 years after IST but that 18% still uses medication for the treatment of AA. In those aged 60 years or older, the probability of being transfusion-independent without alloSCT (T-DFS) was 53% at 5 years, while the probability of being transfusion-independent without any treatment for AA (TT-DFS) was only 34%. These measures are more relevant in clinical practice than separate cumulative incidences of relapse and other failures. Although T-DFS and TT-DFS are lower for older patients compared to younger patients, we do not think that older age should be an absolute contra-indication for ATGAM-based IST in patients with aplastic anemia as suggested by others [6]. In our cohort this treatment is effective and safe as indicated by the occurrence of only one death potentially associated with ATGAM treatment. A possible explanation for this difference between our study and the study of Fattizzo is that in that study patients were treated with different types of ATG including rabbit-derived ATG which is associated with deeper T-cell depletion and potentially more toxicity than the currently used horse-derived ATGAM [21, 22], and that patients were included who were treated decades ago when the supportive therapy including the possibility for anti-fungal prophylaxis was less developed than nowadays. As therapy with ATG is associated with non-lethal side effects and necessitates hospitalization, it can be argued that old and frail patients are preferentially treated with less intensive regimens like cyclosporine with or without Eltrombopag with no need for hospitalization [6, 23]. The long-term treatment success of these therapies should however be compared to the outcomes of ATGAM-based IST.

How the addition of Eltrombopag to first-line IST will improve the enduring treatment success is not yet known. All patients in this analysis received first-line treatment with ATGAM and CsA without the addition of Eltrombopag as this had not been registered for first-line treatment in the Netherlands during the study period. The prospective randomized RACE study showed that the addition of Eltrombopag to this combination leads to an increased complete response rate at 3 months (22% versus 10%). However, this did not translate into a statistical difference in OS at 2 years (90% versus 85%). Furthermore the number of patients that underwent alloSCT as second-line treatment was similar in both treatment arms [9]. Longer follow-up of the RACE study will show whether the addition of Eltrombopag to first-line IST for AA will improve the long-term outcome, preferably by showing a better TT-DFS. In our opinion, in addition to OS, analysis of long-term treatment success of IST should include comprehensive endpoints incorporating the development of MDS or AML and the need for chronic treatment for AA.

In order to predict which adult patients with AA potentially benefit most from first-line treatment with ATGAM-based IST, it is important to identify which baseline factors are associated with long-term treatment success. The detailed clinical data from the Dutch AA registry formed the basis for our multi-state model, which was used to calculate model-based outcomes for different reference patients. We showed that at the start of treatment, lower age, less severe AA and the presence of a PNH-clone of ≥ 1% all increased the probability of reaching the TT-DFS state. For example, patients of 60 years or older with severe AA and no PNH-clone have a poor model-based 5-year TT-DFS (24%, 95%-CI 14–41). On the other hand, patients of 60 years or older with non-severe AA and a PNH-clone of ≥ 1% have a model-based 5-year TT-DFS of 41% (95%-CI 26–65). For both reference patients the model-based 5-year T-DFS, which can still be considered a satisfying outcome, is 17% points higher. Predictions of treatment success may be relevant in the shared decision making whether to start ATG-based IST.

In summary, we introduced Transplantation-, Treatment- and Disease-free survival, as the ultimate goal for patients with AA who are treated with ATGAM-based IST. Based on patients’ age, disease severity and presence of PNH-clone, the probability of reaching this positive outcome can be estimated. If confirmed in another cohort, this could be used as input for personalized treatment decisions.

## Supplementary Information

Below is the link to the electronic supplementary material.

Supplementary Material 1

## Data Availability

The data that support the findings of this study are available from the corresponding author upon reasonable request.
