# Supplementary material for: Anti-thymocyte globulin-based treatment frequently leads to enduring treatment success in both old and young adult patients with aplastic anaemia: a real-world analysis from the Dutch aplastic anaemia registry
Source: Ann Hematol. 2026 Jan 22;105(2):51. doi: 10.1007/s00277-026-06743-5 (PMC12827313; doi:10.1007/s00277-026-06743-5)
Supplement: Supplementary file 1 — Supplementary Material 1 [file 277_2026_6743_MOESM1_ESM.docx]

**Supplementary Methods – guideline and treatment schedule**

Dutch Guidelines for the diagnosis and management of adults with acquired aplastic anemia 2013. Published in Dutch in: Nederlands Tijdschrift voor Hematologie 2013 (6),202-213.

Guideline for treatment

|  | Young patients | Elderly patients |
| --- | --- | --- |
| First line | AlloSCT if a HLA identical sibling is available  IST if no HLA identical sibling is available | IST |
| Second line | AlloSCT with HLA identical unrelated donor  Second-line IST if no HLA identical unrelated donor is available | AlloSCT with an HLA identical sibling or unrelated donor  Second-line IST if no HLA identical unrelated donor is available |
| Third line | Alternative IST  AlloSCT with alternative donor  Supportive care only | Alternative IST  AlloSCT with alternative donor  Supportive care only |

Young patients: below 40 years but in case of severe AA without comorbidities, upper age limit of 50 years can be considered.

For second-line IST Rabbit-derived Thymoglobulin is suggested.

Treatment schedule with IST

| Day | 1 | 2 | 3 | 4 | 5 | 6 |
| --- | --- | --- | --- | --- | --- | --- |
| ATGAM 40 mg/kg |  |  |  |  |  |  |
| Cyclosporin 2.5 mg/kg daily |  |  |  |  |  |  |

Systemic corticosteroids should be given for 1 3 to 4 week period after start of ATGAM to avoid serum sickness.

Aim for Cyclosporin levels between 200 and 300 ug/L, in elderly patients or in case of toxicity levels between 150 and 200 ug/L. In case of a hematological response it is advised to taper Cyclosporin starting at 6 to 12 months after start with 5-10% per month.

**Supplementary Methods – Definitions of different states in the multi-state model.**

Starting state and time of the model is the start of the IST (ATGAM and Cyclosporine). As all patients are also transfusion-dependent at that time, this state is called ‘transfusion-dependent’. If a patient has been transfusion-independent for 4 weeks, this patient will move to the state ‘transfusion-independent with therapy’ after these 4 weeks. If a patient in this state has stopped all non-transplant therapy for 2 weeks and remains transfusion-independent, the patient will move to the state ‘free of transfusion and therapy’. Transfusion-independent patients who become transfusion-dependent and patients who have to restart non-transplant treatment will move to the appropriate states. From these three states, patients can experience three failure events: requirement of alloSCT, start of treatment for another bone marrow disease (AML or MDS) and death. The latter two are absorbing states, implicating that patients can never leave the state. AlloSCT is split into transfusion dependency after alloSCT, transfusion independency after alloSCT and death after alloSCT to evaluate the outcome after alloSCT as non-first-line treatment.

**Supplementary Methods – semi-parametric analyses**

To estimate the effects of patient age, presence of a PNH-clone and severity of the AA on the hazards of becoming transfusion-independent with and without non-transplant-treatment, we used two multivariable Cox proportional hazards regression models for the transition from ‘transfusion-dependent’ to ‘transfusion-independent with therapy’ and the transition from ‘transfusion-independent with therapy’ to ‘free of transfusion and therapy’, and one univariable model for the combined transitions from ‘transfusion-dependent’, ‘transfusion-independent with therapy’ or ‘free of transfusion and therapy’ to ‘death without alloSCT/AML/MDS’. The first and second model included patient age (18-39, 40-59 and 60-80 years), the presence of a PNH-clone (<1% and ≥1% PNH-clone), and AA severity (NSAA, SAA, and VSAA) because of their potential relevance shown in previous studies. The third model included only patient age (18-59 and 60-80 years). The transitions for the third model were combined by assuming a shared baseline hazard because of the low number of events for the separate transitions to death without alloSCT/AML/MDS (n = 1 to 7). Patients for whom data about PNH-clone at baseline was not known were excluded from the semi-parametric analyses. Two-sided p-values <0.05 were considered statistically significant.

**Supplementary Tables**

**Table S1**

Participating hospitals and year in which inclusion of patients in the registry started

| Hospital | Year |
| --- | --- |
| Leiden University Medical Centre  Amsterdam Medical Centre  University Medical Centre Groningen  Antonius Hospital Nieuwegein  Medical Centre Twente  Erasmus Medical Centre Rotterdam  Radboud Medical Centre Nijmegen  University Medical Centre Utrecht  VU Medical Centre  OLVG Hospital  Maastricht University Medical Centre | 2012  2012  2012  2012*  2013  2014  2014  2014  2014  2014**  2017 |

*Hospital stopped treating AA patients with ATG in 2015

**Hospital stopped treating AA patients with ATG in 2014

**Table S2. Causes of death during 5 years after start of IST**

| Cause of death (n) | Time after start of IST in months |
| --- | --- |
| Complications of alloSCT as second-line treatment (5)  MDS/AML (3)  Pneumonia (3)  Other malignancy (3)  Hemorrhage (2)  Refractory aplastic anemia (2)  Cardiac failure (1)  Abdominal aneurysm (1)  Unknown (1) | 8, 10, 12, 16, 25  8, 45, 50  11, 20, 32  1, 3, 24  1, 37  32, 59  0.3  9  40 |

**Table S3. Model-based 5-year T-DFS and TT-DFS for all possible reference patients**

| Reference patient* | T-DFS | TT-DFS |
| --- | --- | --- |
| 18-39, ≥1% PNH-clone, NSAA | 0.77 (0.40-1.00) | 0.60 (0.41-0.88) |
| 40-59, ≥1% PNH-clone, NSAA | 0.78 (0.68-0.90) | 0.55 (0.38-0.79) |
| 60-80, ≥1% PNH-clone, NSAA | 0.58 (0.44-0.77) | 0.41 (0.26-0.65) |
| 18-39, <1% PNH-clone, NSAA | 0.79 (0.69-0.90) | 0.57 (0.40-0.79) |
| 40-59, <1% PNH-clone, NSAA | 0.71 (0.61-0.83) | 0.47 (0.31-0.71) |
| 60-80, <1% PNH-clone, NSAA | 0.54 (0.40-0.71) | 0.35 (0.22-0.56) |
| 18-39, ≥1% PNH-clone, SAA | 0.78 (0.69-0.90) | 0.55 (0.40-0.75) |
| 40-59, ≥1% PNH clone, SAA | 0.70 (0.60-0.83) | 0.45 (0.29-0.69) |
| 60-80, ≥1% PNH-clone, SAA | 0.53 (0.39-0.71) | 0.34 (0.21-0.54) |
| 18-39, <1% PNH-clone, SAA | 0.72 (0.61-0.84) | 0.47 (0.33-0.68) |
| 40-59, <1% PNH-clone, SAA | 0.54 (0.42-0.70) | 0.31 (0.18-0.56) |
| 60-80, <1% PNH-clone, SAA | 0.41 (0.29-0.59) | 0.24 (0.14-0.41) |
| 18-39, ≥1% PNH-clone, VSAA | 0.78 (0.68-0.90) | 0.59 (0.41-0.84) |
| 40-59, ≥1% PNH-clone, VSAA | 0.71 (0.61-0.84) | 0.50 (0.33-0.75) |
| 60-80, ≥1% PNH-clone, VSAA | 0.54 (0.40-0.72) | 0.37 (0.23-0.59) |
| 18-39, <1% PNH-clone, VSAA | 0.73 (0.62-0.85) | 0.52 (0.35-0.77) |
| 40-59, <1% PNH-clone, VSAA | 0.56 (0.43-0.74) | 0.36 (0.21-0.62) |
| 60-80, <1% PNH-clone, VSAA | 0.43 (0.30-0.61) | 0.27 (0.16-0.46) |

*Some combinations of characteristics are scarcely present in the data.

**Supplementary Figures**

**
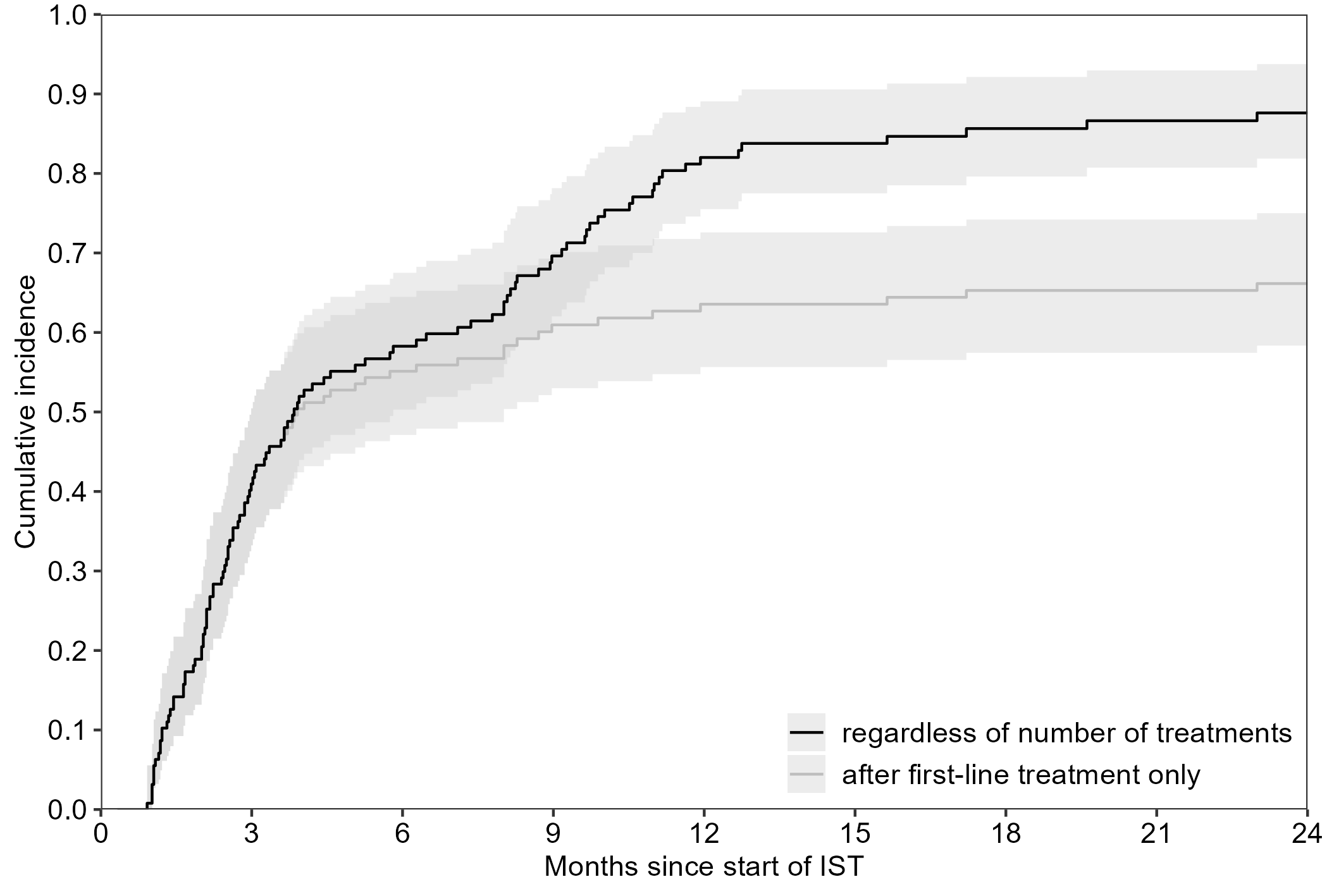
**

**Supplemental Figure 1. Cumulative incidence of achievement of transfusion independency during the first 2 years.** The two curves with associated 95% confidence intervals were estimated in separate competing risks models. The first model considered death and treatment for AML or MDS as competing events (black curve), the second model also considered start of any second-line treatment as competing event (grey curve).

**
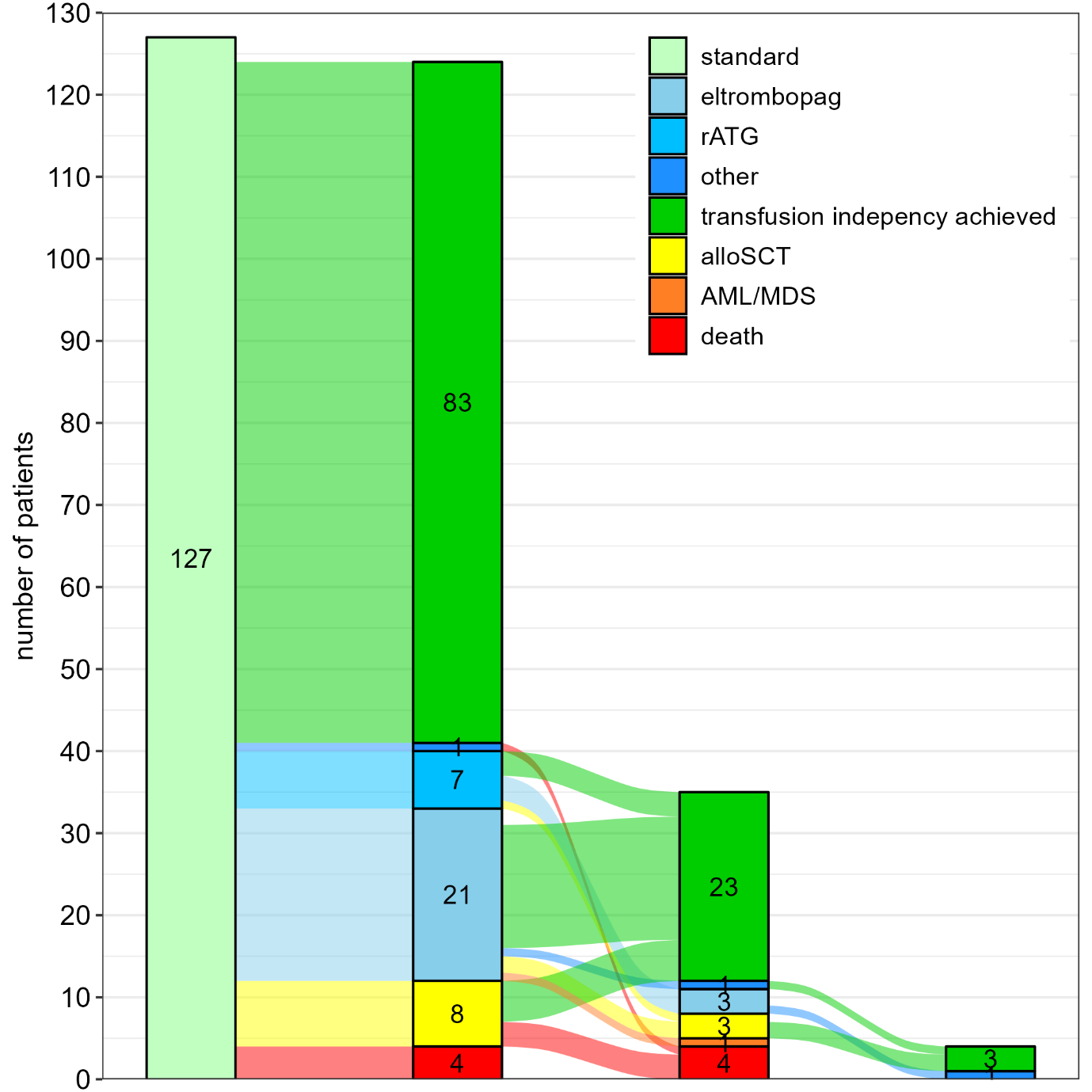
**

**Supplemental Figure 2. Overview of the different lines of treatment until the first achievement of transfusion independency during the first 2 years.** All patients started with the standard therapy consisting of ATG and ciclosporin. Each new column represents addition of another line of treatment, achievement of transfusion independency or occurrence of a clinical event. For example, 83 patients became transfusion-independent without any other treatment. Eltrombopag was started in 21 transfusion-dependent patients, after which 15 became transfusion-independent. 8 patients received alloSCT as second-line treatment, of whom 3 died. Death following Acute Myeloid Leukemia or Myelodysplastic Syndrome is ignored. After alloSCT, only death and achievement of transfusion independency are considered.

**
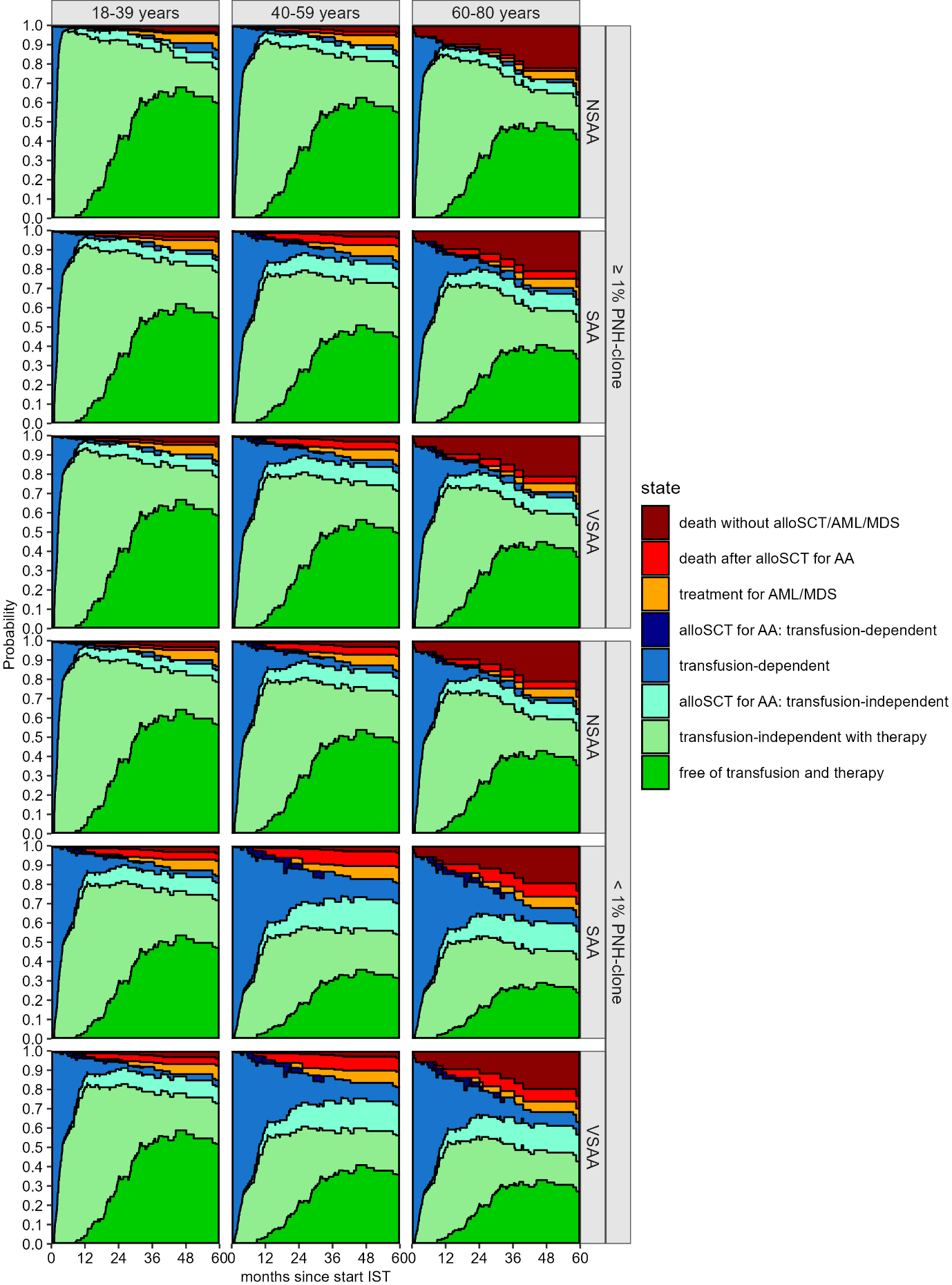
**

**Supplemental Figure 3. Model-based outcomes for reference patients with all possible combinations of characteristics**. Based on the multi-state model in Figure 1 and the transition-specific Cox models in Table 2. The difference between two adjacent curves represents the probability of being in the corresponding state. See Supplemental Table 3 for the model-based T-DFS and TT-DFS at 5 years after start of IST. Note that some combinations of characteristics are scarcely present in the data.
